# Brain structural differences between 73- and 92-year olds matched for childhood intelligence, social background, and intracranial volume

**DOI:** 10.1016/j.neurobiolaging.2017.10.005

**Published:** 2018-02

**Authors:** Stuart J. Ritchie, David Alexander Dickie, Simon R. Cox, Maria del C. Valdés Hernández, Ruth Sibbett, Alison Pattie, Devasuda Anblagan, Paul Redmond, Natalie A. Royle, Janie Corley, Susana Muñoz Maniega, Adele M. Taylor, Sherif Karama, Tom Booth, Alan J. Gow, John M. Starr, Mark E. Bastin, Joanna M. Wardlaw, Ian J. Deary

**Affiliations:** a Department of Psychology, The University of Edinburgh, Edinburgh, UK; b Centre for Cognitive Ageing and Cognitive Epidemiology, The University of Edinburgh, Edinburgh, UK; c Institute of Cardiovascular and Medical Sciences, College of Medical, Veterinary & Life Sciences, University of Glasgow, Glasgow, UK; d Brain Research Imaging Centre, the University of Edinburgh, Edinburgh, UK; e Scottish Imaging Network, A Platform for Scientific Excellence (SINAPSE) Collaboration; f Centre for Clinical Brain Sciences, The University of Edinburgh, Edinburgh, UK; g Alzheimer Scotland Dementia Research Centre, The University of Edinburgh, Edinburgh, UK; h Department of Neurology and Neurosurgery, McConnell Brain Imaging Center, Montreal Neurological Institute, McGill University, Montreal, Quebec, Canada; i Department of Psychiatry, Douglas Mental Health University Institute, McGill University, Verdun, Quebec, Canada; j Department of Psychology, Heriot-watt University, Edinburgh, UK

**Keywords:** Aging, Structural MRI, Brain volume, White matter hyperintensities, Lesion mapping

## Abstract

Fully characterizing age differences in the brain is a key task for combating aging-related cognitive decline. Using propensity score matching on 2 independent, narrow-age cohorts, we used data on childhood cognitive ability, socioeconomic background, and intracranial volume to match participants at mean age of 92 years (*n* = 42) to very similar participants at mean age of 73 years (*n* = 126). Examining a variety of global and regional structural neuroimaging variables, there were large differences in gray and white matter volumes, cortical surface area, cortical thickness, and white matter hyperintensity volume and spatial extent. In a mediation analysis, the total volume of white matter hyperintensities and total cortical surface area jointly mediated 24.9% of the relation between age and general cognitive ability (tissue volumes and cortical thickness were not significant mediators in this analysis). These findings provide an unusual and valuable perspective on neurostructural aging, in which brains from the 8th and 10th decades of life differ widely despite the same cognitive, socioeconomic, and brain-volumetric starting points.

## 1 Introduction

Many changes in brain structure occur during normal aging. Understanding and characterizing these age-related differences are important because they have been linked to aging-related cognitive decline, a pervasive phenomenon with a substantial predicted effect on aging societies (Brayne, 2007, Luengo-Fernandez et al., 2010). A fuller understanding of later-life brain changes will aid in the search for interventions to ameliorate this decline (Raz and Lindenberger, 2013). Relatively few studies have modeled both brain and cognitive age differences, and fewer have included participants over the age of 90 years (Dickie et al., 2013). In the present study, we quantified age differences in a variety of neurostructural measures using an unusual design: we compared closely matched participants from 2 independent narrow-aged samples in later life, 1 aged 73 years and the other 92 years. We then tested the extent to which neuroanatomical differences could explain the large age-related cognitive differences between the 2 samples.

The most-studied neuroanatomical measure with reference to aging is brain volume. Volume peaks in early adulthood, before a period of relatively mild decline through midlife, and more rapid degeneration in older age (Fjell and Walhovd, 2010). In nonpathological aging, adults aged more than 60 years experience around a 0.5% decline in total brain volume per year (Fotenos et al., 2005), with volumetric declines seen in both gray matter and white matter, in regions across the entire brain (Dickie et al., 2015, Giorgio et al., 2010, Kruggel, 2006, Raz et al., 2005, Walhovd et al., 2011, Ziegler et al., 2011). Cortical surface area follows a similar trajectory of decline (Hogstrom et al., 2013), and most regions of the brain exhibit cortical thinning with age, with the loss of up to ∼0.6 mm of cortical thickness per decade (Thambisetty et al., 2010; see also Fjell et al., 2009; Shaw et al., 2016a, Shaw et al., 2016b). Finally, the volume of white matter hyperintensities (WMHs) tends to increase with advancing age (Morris et al., 2009, Ritchie et al., 2015a). These hyperintensities, which are commonly seen on fluid-attenuated inversion recovery (FLAIR) brain magnetic resonance imaging (MRI) scans of older people and vary in their extent between individuals, are indicators of pathology thought to be related to small vessel disease, though debate continues on their precise etiology (see Wardlaw et al., 2015, for detailed discussion).

Deteriorations in the above-listed brain measures have been linked, in longitudinal studies, to declines in key cognitive faculties such as fluid intelligence, reasoning, mental speed, and memory, which decline on average throughout adulthood (Salthouse, 2004). For example, Schmidt et al. (2005) showed that declining brain volume was related to loss of cognitive skills such as memory and visuopractical abilities (see also Jokinen et al., 2012, Persson et al., 2016, Ritchie et al., 2015a). In a meta-analysis, Kloppenborg et al. (2014) showed that advancing WMH levels were related to decrements in all measured cognitive abilities.

There is relatively little evidence on which of these neuroanatomical variables are the most relevant for explaining cognitive aging, since few studies have analyzed multiple imaging variables simultaneously. In a previous study of one of the cohorts involved in the present analysis (Ritchie et al., 2015b), we measured multiple neuroanatomical measures and related them to a broad latent variable of general cognitive ability (so-called “*g*”) measured at the age of 73 years. Total brain volume made the largest contributions to explaining variance in *g*, but other variables such as WMH and cortical thickness made additional, incremental contributions (see also Kievit et al., 2012). Thus, it is likely that several different aspects of brain structure are independently relevant to understanding the aging of cognitive abilities. However, these studies focus on cognitive ability level, rather than the age-related differences in these abilities.

In testing the extent to which brain structure can account for age differences in cognitive functioning, the present study took the approach of Kievit et al. (2014). They used structural equation model–based mediation analysis to test whether the age variance in cognitive ability could, in part, be explained by different neuroanatomical measures. They showed, for instance, that fractional anisotropy of the forceps minor and the volume of Brodmann area 10 were parallel mediators (explaining 18.2% in total) of the association between age and fluid intelligence in a sample with an age range of 18–89 years. Although the selection of brain regions included in that analysis was limited (2 cortical regions and 2 tracts), their results contributed to our understanding of the multifaceted nature of brain aging and its relation to key cognitive outcomes. That, in addition to a detailed characterization of aging across various brain imaging measures, was the aim of the present study.

### 1.1 The present study

Here, we extensively characterized whole and regional brain differences between 2 narrow-age cohorts of older people, 1 aged around 73 years and the other around 92 years. Unusually, both cohorts had data available on the same well-validated general cognitive ability test taken at the age of 11 years, as well as retrospective data on their socioeconomic status from childhood and adulthood. We used propensity score matching on these background variables, as well as on a measure of maximal brain size (their intracranial volume), to reduce confounding in the comparison of the 2 cohorts in later life. Because socioeconomic and early cognitive differences may influence the intercept (if not necessarily the slope) of aging-related changes (e.g., Barulli and Stern, 2013, Tucker-Drob et al., 2009), it was important to compare participants who have been matched on these variables.

Using these well-matched cohorts, we ran the following 3 analyses. First, we characterized the extent of the 19-year age differences in multiple broad brain volumetric measures: total brain volume, gray and white matter volumes, and the volume of WMHs. Second, we examined the gray matter in more detail, using parcellation to map volume and surface area differences in each of 54 gray matter regions of interest between the 73- and 92-year olds. We also used a vertex-wise method to examine the cohort differences in cortical thickness across the entire brain. Third, we used mediation analyses to test whether differences in *g* (indicated by the same 3 cognitive tests taken by both cohorts) between the samples could be accounted for by differences in brain structure.

## 2 Method

### 2.1 Participants

Members of both the Lothian Birth Cohort 1921 (LBC1921; Deary et al., 2004b) and the Lothian Birth Cohort 1936 (LBC1936; Deary et al., 2007, Deary et al., 2012) studies were included in the present analysis. Both cohorts are studies of aging that follow up individuals who, at the age of 11 years, took part in the Scottish Mental Survey of 1932 or 1947. The cohorts have been followed up at multiple waves in later life; for the present study, we focus on data from the fifth wave of the LBC1921 (total *n* = 59, mean age = 92.1 years, standard deviation [SD] = 0.34) and the second wave of the LBC1936 (total *n* = 866, mean age = 72.5 years, SD = 0.71). At these waves, *n* = 53 members of the LBC1921 and *n* = 731 members of the LBC1936 attended for a structural MRI scan (as described below, the final matched sample involved *n* = 42 LBC1921 members and *n* = 126 LBC1936 members). In the LBC1921 cohort, cognitive/medical testing and brain scanning were completed on the same day in all but a few cases (mean gap = 0.04 days, SD = 0.27). In the LBC1936, the participants all made 2 separate visits (mean gap = 65.04 days, SD = 39.57).

Approval for the LBC1921 study was obtained from the Lothian Research Ethics Committee (wave 1: LREC/1998/4/183; wave 3: 1702/98/4/183) and the Scotland A Research Ethics Committee (waves 4 and 5: 10/S1103/6). Approval for the LBC1936 study was obtained from the Multicentre Research Ethics Committee of Scotland (wave 1: MREC/01/0/56), the Lothian Research Ethics Committee (wave 1: LREC/2003/2/29), and the Scotland A Research Ethics Committee (waves 2 and 3: 07/MRE00/58). All participants provided written, informed consent before any measurements were taken.

### 2.2 Measures

#### 2.2.1 Brain MRI acquisition and volumetric processing

Brain MRI acquisition parameters were described in detail for the LBC1936 by Wardlaw et al. (2011). All subjects (from both cohorts) had brain MRI on the same 1.5T GE Signa Horizon HDx clinical scanner (General Electric, Milwaukee, WI, USA), maintained on a careful quality assurance program. Both cohorts underwent the same structural imaging examination consisting of high-resolution three-dimensional T1-weighted volume and T2-, T2*-, and FLAIR-weighted sequences. The voxel resolutions of the T2-, T2*-, and FLAIR-weighted MRI sequences were 2, 2, and 4 mm^3^ for the LBC1936 and 2.2, 2.2, and 4.4 mm^3^ for the LBC1921; the voxel resolution for the three-dimensional T1-weighted volume scan (1.3 mm^3^) was identical between cohorts.

We measured intracranial, whole brain, gray matter, normal-appearing white matter, and WMH volumes in cubic centimeter using a validated multispectral image processing method that combines T1-, T2-, T2*-, and FLAIR-weighted MRI sequences for segmentation (Valdés Hernández et al., 2010). All sequences were coregistered, and tissue volumes were estimated by cluster analysis of voxel intensities. We explicitly defined WMH as punctate, focal, or diffuse lesions in all subcortical regions and manually edited WMH masks according to STandards for ReportIng Vascular Changes on NEuroimaging guidelines (Wardlaw et al., 2013). Editing was overseen by a consultant neuroradiologist (author JMW). We manually checked these segmented images for accuracy blinded to all clinical details, corrected errors, and excluded imaging-detected infarcts from WMH volumes (Wang et al., 2012). Previous research has shown good interrater reliability using these methods (low SDs of voxels between raters; see Table 4 in Valdés Hernández et al., 2010). We tested the correlation of the overall gray matter volume produced using this method with the overall gray matter estimate derived using FreeSurfer, which we used for our cortical subregional analysis (see below). Across the participants included in the present study, this correlation was *r*(164) = 0.89.

#### 2.2.2 Lesion distribution maps

To produce a map of the distribution of WMH across the brain (as per Valdés Hernández et al., 2015), we coregistered all WMH masks to a common normal-aging brain template (Dickie et al., 2016a) using affine registration on FSL-FLIRT (Jenkinson et al., 2002), averaged all the coregistered WMH masks of the data sets from each cohort (i.e., from LBC1921 and LBC1936), and generated 2 probability distribution maps of WMHs. Then, we mapped both probability maps in the template to analyze the spatial distribution of WMHs from each sample. Illustrations of the distributions were produced in Mango v4.0 (http://ric.uthscsa.edu/mango/).

#### 2.2.3 Subregional volumes and surface areas

FreeSurfer v5.3 (http://surfer.nmr.mgh.harvard.edu/) was used for cortical reconstruction and segmentation of T1-weighted volumes according to the Desikan-Killiany parcellation protocol (Desikan et al., 2006). Briefly, the steps involved removal of non-brain tissue, intensity normalization, tessellation of the white/gray matter boundary, and inflation and registration of the cortical surface to the spherical atlas according to the folding patterns of each individual (Fischl and Dale, 2000, Fischl et al., 2004, Segonne et al., 2004, Segonne et al., 2007). This yielded the volume and surface area of 54 cortical regions of interest, where the surface area represented the sum of all triangular tessellations in each anatomical regions at the midpoint between gray matter and white matter. All outputs were visually inspected for processing failures or deficiencies (including tissue identification and boundary positioning errors). Segmentation failed in 3 cases in the LBC1921 (leaving *n* = 39 for the volume and surface area comparisons) and 4 cases in the LBC1936 (leaving *n* = 122). A small proportion of regions of interest (8.45% and 1.64% for LBC1921 and LBC1936 cohorts, respectively) were affected by tissue identification or boundary positioning errors and were excluded from the analyses. Therefore, the total N (across both cohorts) for region of interest–based analyses ranged from 153 to 161 (M = 159, SD = 1.75). Cortical surface visualizations used the freely available Liewald-Cox Heatmapper tool (http://www.ccace.ed.ac.uk/research/software-resources/software).

#### 2.2.4 Cortical thickness measurement

Cortical thickness was measured using the fully automated CIVET v1.1.12 image processing pipeline developed at the Montreal Neurological Institute (Ad-Dab'bagh et al., 2006,; Zijdenbos et al., 2002). CIVET measures cortical thickness at 81,924 vertices (the perpendicular distance between gray and white matter surfaces) across the cortex (Ducharme et al., 2016, Karama et al., 2009, Karama et al., 2015). For clarity, we use “vertex” to refer to the perpendicular distance between gray and white matter surfaces, not the cranial vertex. Cortical surface maps from each subject were manually inspected according to previously validated standards (Ducharme et al., 2016). Approximately 10% of subjects from each full cohort failed cortical thickness processing because of poor scan quality or motion artifacts. Interrater reliability for passing or failing quality control was 0.93 in the article by Ducharme et al, and one of the original raters of that article (author SK) rated the cortical thickness outputs here. Of the final matched samples, after this quality control procedure 5 subjects from the LBC1921 had missing data (leaving a final *n* = 37 for the cortical thickness analysis), along with 14 subjects from the LBC1936 (leaving a final *n* = 112).

Cortical vertex-wise regression analyses were performed using the SurfStat MATLAB toolbox (http://www.math.mcgill.ca/keith/surfstat). The statistical significance of results for cortical thickness was corrected for multiple comparisons using random field theory (RFT) to avoid false positives when more than 80,000 tests were performed (Chung et al., 2005). RFT identifies statistically significant “clusters” of vertices and vertex “peaks”. Cluster *p*-values show regions of connected vertices with *p*-values below 0.001 in clusters whose extent is significant at *p* < 0.05 (http://www.math.mcgill.ca.keith/surfstat), that is, a collection of connected vertices with *p* < 0.001 that was unlikely to occur by chance. Vertex *p*-values show individual vertices where individual *t* scores are above the vertex-wise RFT critical *t*-value that is derived via the expected Euler characteristic (EC ≈ critical *p*-value [0.05]) and number of resolution elements (“resels”) in the *t* cortical map (Brett et al., 2004, Worsley et al., 2004).

#### 2.2.5 Matching variables

We ensured that the cohorts were comparable by matching them on 5 variables that indexed their social, cognitive, and neural background (the propensity score matching procedure is described in the “Statistical Analysis” section, below). First, as noted previously, all participants in both cohorts had childhood cognitive testing data available from age 11 years based on the same test. The test used, the Moray House Test (MHT) No. 12 (Scottish Council for Research in Education, 1933), assesses a variety of cognitive functions with an emphasis on verbal reasoning, and is strongly correlated with other commonly-used tests of general cognitive function in childhood and later life (Deary et al., 2004b). Second, each participant's father's socioeconomic status when the participant was aged 11 years was collected by questionnaire around the time of the first wave of each study (at about 79 years for LBC1921 participants and 70 years for LBC1936 participants) and classified using an index of their occupational class on a scale of I (professional) to V (unskilled; General Register Office, 1956). For female cohort members, the highest occupational class of the household was taken. Third, information on the participants' own socioeconomic status was collected during the same interview; it was then classified, based on their most prestigious occupation they held before retirement, on the same scale for the LBC1921 participants and a similar scale for the LBC1936 (Office of Population Censuses and Surveys, 1980). Fourth, we matched the participants for intracranial volume, a proxy measure of maximal lifetime brain size. Intracranial volume, unlike total brain tissue volume (which shows steep declines across adulthood), has been shown to be highly stable between ages 18 and 96 years (see Fig. 2 in Royle et al., 2013). Its measurement in the present samples is described previously. Fifth, we matched the participants for sex, such that the sex ratio within the matched sample of each cohort was similar.

**Fig. 1:**
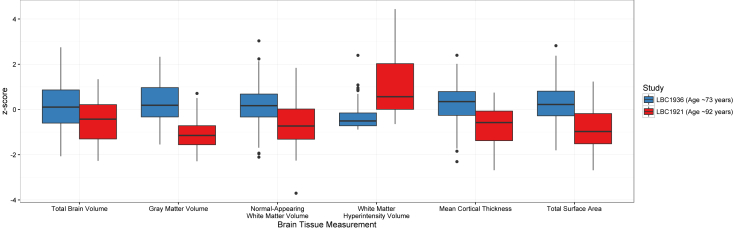
Brain tissue differences between the LBC1936 (age ∼73 years) and the LBC1921 (age ∼92 years) for each tissue type, on a standardized (*z*) scale. Each box of the “boxplot” displays the median (dark central line) and the 25% and 75% quantiles (lower and upper “hinges” on each box, respectively), with “whiskers” extending to the highest (upper whisker) or lowest (lower whisker) observation that was no further than 1.5 multiplied by the interquartile range away from the box's “hinge”. Outlying values beyond the whiskers are shown as dots. *p*-values for all cohort comparisons were <0.001 (see Table 1). Abbreviations: LBC1921, Lothian Birth Cohort 1921; LBC1936, Lothian Birth Cohort 1936.

#### 2.2.6 Later-life cognitive tests

Members of both cohorts completed multiple cognitive tests in later life. For the purposes of this study, we took 3 tests that were administered in exactly the same setting in both cohorts and that could be expected to decline with age (i.e., they measured “fluid”-type aspects of cognitive ability; see Salthouse, 2004). The first test was Digit-Symbol Substitution from the Wechsler Adult Intelligence Scale, Third UK Edition (WAIS-III^UK^; Wechsler, 1998a), a measure of cognitive processing speed. The second was the Logical Memory test (story A only) from the Wechsler Memory Scale–Revised in the LBC1921 (WMS-R; Wechsler, 1987) and the Wechsler Memory Scale, Third UK Edition in the LBC1936 (WMS-III^UK^; Wechsler, 1998b), a test of verbal declarative memory. The third was a phonemic Verbal Fluency test (using the letters C, F, and L, for 1 minute each; Lezak, 2004), which taps an aspect of executive function. We used structural equation modeling (see below) to estimate a general (*g*) factor of cognitive ability from these 3 tests.

Each participant was also administered the Mini–Mental State Examination (MMSE; Folstein et al., 1975), a dementia screening instrument that assesses basic aspects of language, attention, and orientation to place and time, with a maximum score of 30. Scores below 24 are commonly considered indicative of possible mild cognitive impairment or dementia.

#### 2.2.7 Health variables

A variety of self-reported (categorical) and blood-derived (continuous) health variables were assessed identically in both cohorts. The self-reported variables included smoking status (never, ex-, or current), self-rated health both at present (from poor to excellent) and compared to 1 year ago (much better to much worse), and diagnoses of a variety of conditions including hypertension, diabetes, cardiovascular disease, and stroke. The blood-derived biomarkers consisted of blood hemoglobin, white cell count, fibrinogen, d-dimer, estimated glomerular filtration rate, and glycated hemoglobin.

### 2.3 Statistical analysis

#### 2.3.1 Propensity score matching

The main aim of the study was to compare brains of young-old and old-old people who had similar cognitive, social, and brain-volumetric starting points. Participants were matched across the 2 cohorts using the 5 matching variables described previously (childhood MHT cognitive score, father's social class, the participant's own achieved social class, intracranial volume, and sex). To do this, we used propensity score matching in the “nonrandom” package for R (Stampf, 2014). There were 42 LBC1921 participants who had full data on all the matching variables and who had attended for an MRI scan; for each of these, we allowed the matching process to select 3 LBC1936 participants who also had all matching variables available and attended scanning (thus 126 LBC1936 participants). Participants were matched if they were within 0.2 SD of the logit of the propensity score produced from a simultaneous logistic regression model including all 5 matching variables. We ensured that the matching was adequate by comparing across the samples on each of the matching variables using Welch's 2-sample *t*-tests, calculating Cohen's *d*_*s*_ for the standardized effect size, as described by Lakens (2013). The same procedure was used to compare the samples on each of the brain variables of interest.

#### 2.3.2 Mediation analysis

Another aim of the study was to test the extent to which general fluid cognitive differences between young-old and old-old people are accounted for by brain structural differences. We used structural equation modeling in the “lavaan” package for R (Rosseel, 2012) to perform mediation analysis. The predictor variable was the cohort to which each participant belonged (i.e., a proxy for whether they were 73 or 92 years old), the outcome variable was the *g*-factor of cognitive ability estimated from the 3 cognitive tests, and the potential mediator variables were the brain measures.

First, we tested whether the *g*-factors within each cohort were comparable, by testing their cross-cohort measurement invariance as described by Widaman et al. (2010), as shown in Table S1. There were no significant differences, by *χ*^2^ test, between models with configural, weak, strong, and strict measurement invariance. There were negligible differences in the Akaike information criterion, whereas the Bayesian information criterion favored the models with stricter factorial invariance. Thus, we considered the construct of *g* to be comparable across the cohorts and proceeded with the mediation analyses.

We tested whether the paths from cohort to *g* (the “direct” path), from cohort to the mediator (the first part of the “indirect” path), and from the mediator to *g* (the second part of the “indirect” path) were significant, using bias-corrected and accelerated 95% confidence intervals (CIs) (DiCiccio and Efron, 1996) bootstrapped 1000 times, to test whether the mediation (the product of the 2 “indirect” paths) was statistically significant. As an effect size, we calculated a “percentage of mediation” (Iacobucci et al., 2007), estimating how much the “direct” path was attenuated by the inclusion of the “indirect” path. We tested whether multiple mediators—for instance, gray matter, normal-appearing white matter, and WMH volumes—were incrementally significant mediators in a simultaneous model.

## 3 Results

### 3.1 Matching and health variables

We first matched the participants across the 2 cohorts using the propensity score procedure described above (the coefficients from the logistic regression used to generate the propensity score are provided in Table S2). To confirm the effectiveness of the propensity score matching procedure, we tested whether there were any significant differences in the matching variables. The results are shown in the upper section of Table 1. Differences in age-11 cognitive ability, background and own-attained social class, and intracranial volume were small and nonsignificant (all *d*_*s*_ values <0.17, all *p*-values > 0.36). The sex ratio did not differ between cohorts (*χ*^2^ ≈ 0.00, *p* = 1.00). This confirmed that there were at most small, nonsignificant differences in these variables between the LBC1921 and LBC1936 participants.

**Table 1:** Comparisons between LBC1921 (*n* = 42; age ∼92 years) and propensity-score-matched LBC1936 (*n* = 126; age ∼73 years) participants on each brain measure, for those who had data on all matching variables

Measure category	Measure	*N*		Sample mean (SD)/%		Difference test		
		LBC1921	LBC1936	LBC1921	LBC1936	*T*	*p*	*d*_*s*_
Matching variables	Father's SES	42	126	2.90 (1.28)	2.87 (1.00)	0.15	0.88	0.03
	Achieved SES	42	126	2.26 (0.99)	2.41 (0.80)	−0.89	0.37	0.16
	Age-11 MHT	42	126	47.36 (10.33)	48.84 (12.45)	−0.76	0.45	0.14
	ICV	42	126	1490.91 (140.18)	1478.37 (141.80)	0.50	0.62	0.09
	Sex	42	126	40.5% male	39.7% male	-	1.00	-
Brain tissue measures	TBV (cm^3^)	38	126	954.73 (95.17)	1015.49 (92.43)	−3.49	<0.001	0.62
	GMV (cm^3^)	37	126	404.76 (39.02)	480.20 (48.20)	−9.77	<0.001	1.74
	NAWM (cm^3^)	37	126	434.02 (70.73)	491.69 (54.05)	−4.58	<0.001	0.82
	WMH volume (cm^3^)	40	126	46.87 (30.42)	13.01 (12.01)	6.81	<0.001	1.21
	MCT (mm)	37	112	2.97 (0.14)	3.13 (0.15)	−6.08	<0.001	1.08
	TSA (cm^2^)	39	122	1377.11 (164.61)	1572.46 (145.77)	−4.74	<0.001	1.12
Later-life cognitive tests	Digit-Symbol	34	125	33.32 (10.66)	58.39 (11.75)	−11.89	<0.001	2.12
	Logical Memory	42	126	9.90 (4.48)	16.70 (3.77)	−8.85	<0.001	1.58
	Verbal Fluency	42	126	38.43 (13.64)	43.77 (12.00)	−2.26	0.02	0.40
Dementia screening	MMSE	42	125	26.90 (2.36)	28.98 (1.29)	−5.45	<0.001	0.97

Differences calculated using Welch's 2-sample *t*-test; for Cohen's *d*_*s*_, see Lakens (2013).

Key: GMV, gray matter volume; ICV, intracranial volume; LBC1921, Lothian Birth Cohort 1921; LBC1936, Lothian Birth Cohort 1936; MCT, mean cortical thickness; MHT, Moray House Test; MMSE, Mini–Mental State Examination; NAWM, normal-appearing white matter volume; SD, standard deviation; SES, socioeconomic status; TBV, total brain volume; TSA, total cortical surface area; WMH, white matter hyperintensity.

Table 2, Table 3 compare both samples across multiple health variables. Compared with the matched younger LBC1936 sample, the older LBC1921 sample rated their health “compared to 1 year ago” more poorly (*χ*^2^ = 22.15, *p* < 0.001) and had higher rates of hypertension (76.2% vs. 42.1%, *χ*^2^ = 13.34, *p* < 0.001) and cancer/tumors (31.0% vs. 12.7%, *χ*^2^ = 7.35, *p* = 0.01). The older sample also had lower levels of blood hemoglobin (124.43 g/L vs. 139.49 g/L; *t* = −6.79, *p* < 0.001, *d*_*s*_ = 1.21; consistent with previous research, e.g., Salive et al., 1992) and estimated glomerular filtration rate (55.63 vs. 63.84 mL/min; *t* = −4.14, *p* < 0.001, *d*_*s*_ = 0.74). There were no significant differences between the cohorts in stroke prevalence (*p* = 0.13) or on any of the other health indicators (all *p*-values > 0.50). No participants in either cohort had a self-reported diagnosis of dementia.

**Table 2:** Comparisons between the LBC1921 (age ∼92 years) and the LBC1936 (age ∼73 years) on categorical health variables

Measure	Frequencies/percentages		*χ*^2^	*p*-value
	LBC1921 (*n* = 42)	LBC1936 (*n* = 126)		
Smoking status	Never smoked = 23	Never smoked = 58	–	0.57
	Ex-smoker = 16	Ex-smoker = 59		
	Current smoker = 3	Current smoker = 9		
Self-reported health	Poor = 2	Poor = 1	–	0.19
	Fair = 5	Fair = 8		
	Good = 16	Good = 40		
	Very good = 16	Very good = 62		
	Excellent = 3	Excellent = 15		
Self-reported health compared to 1y ago	Much worse than 1 y ago = 4	Much worse than 1 y ago = 0	–	<0.001
	Somewhat worse than 1 y ago = 16	Somewhat worse than 1 y ago = 27		
	About the same as 1 y ago = 22	About the same as 1 y ago = 81		
	Somewhat better than 1 y ago = 0	Somewhat better than 1 y ago = 13		
	Much better than 1 y ago = 0	Much better than 1 y ago = 5		
Hypertension	76.2%	42.1%	13.34	<0.001
Diabetes	2.4%	8.7%	1.08	0.30
Hypercholesterolemia	38.1%	43.7%	0.20	0.65
Cardiovascular disease	50.0%	33.3%	3.06	0.08
Leg pain	54.8%	40.5%	2.06	0.15
Blood circulation problems	16.7%	22.2%	0.30	0.58
Stroke	11.9%	7.1%	4.03	0.13
Cancer/tumour	31.0%	12.7%	7.35	0.01
Thyroid disorder	14.3%	12.7%	0.00	1.00
Parkinson's disease	0.0%	0.8%	0.00	1.00
Dementia	0.0%	0.0%	0.00	1.00
Arthritis	42.9%	42.9%	0.00	1.00

*p*-values from Fisher's exact test (initial 3 rows) or from *χ*^2^ test with Yates's continuity correction (remaining rows). One LBC1921 participant who was unsure if he/she had been diagnosed with stroke was classified as not having such a diagnosis for this table.

Key: LBC1921, Lothian Birth Cohort 1921; LBC1936, Lothian Birth Cohort 1936.

**Table 3:** Comparisons between the LBC1921 (age ∼92 years) and the LBC1936 (age ∼73 years) on continuous health (biomarker) measures

Measure	Sample mean (SD)		Difference test		
	LBC1921 (*n* = 42)	LBC1936 (*n* = 126)	*t*	*p*	*d*_*s*_
Blood hemoglobin (g/L)	124.43 (11.95)	139.49 (13.48)	−6.79	<0.001	1.21
White cell count (×10^9^/L)	7.01 (2.05)	6.60 (1.47)	1.18	0.24	0.21
Fibrinogen (g/L)	3.16 (0.76)	3.33 (0.66)	−1.24	0.22	0.22
D-dimer (ng/dL)	376.91 (462.24)	206.71 (197.71)	1.98	0.055	0.35
eGFR (mL/min)	55.63 (12.33)	63.84 (3.93)	−4.14	<0.001	0.74
HbA1c (DCCT)	5.61 (0.40)	5.74 (0.59)	−1.58	0.12	0.28

Differences calculated using Welch's 2-sample *t*-test; *d*-values are Cohen's *d*_*s*_ (see Lakens, 2013).

Key: eGFR, estimated glomerular filtration rate; HbA1c, glycated hemoglobin; LBC1921, Lothian Birth Cohort 1921; LBC1936, Lothian Birth Cohort 1936; SD, standard deviation.

### 3.2 Brain measure comparisons

There were substantial differences between the 2 cohorts in brain volume, gray matter volume, normal-appearing white matter volume, WMH volume, mean cortical thickness, and total cortical surface area (Table 1). The age-92 LBC1921 had significantly and substantially lower volumes than the age-73 LBC1936 on all healthy brain tissue measures (all *p*-values < 0.001), with effect sizes (Cohen's *d*_*s*_) between 0.62 and 1.74. The older LBC1921 participants had significantly and substantially higher volumes of WMHs (*p* < 0.001, *d*_*s*_ = 1.21). The standardized differences are illustrated in Fig. 1.

**Fig. 2:**
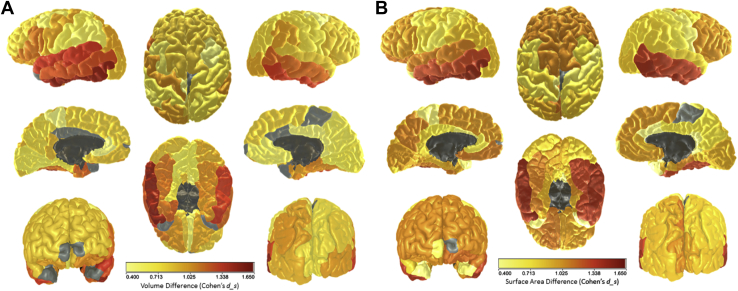
Differences between the LBC1936 (age ∼73 years) and the LBC1921 (age ∼92 years) in (A) mean volume and (B) mean surface area across parcellated subregions. Gray indicates null difference. For full numerical results for volume and surface area, respectively, see Tables S3 and S4. Light gray denotes nonsignificant associations, whereas dark gray denotes unlabeled regions. Abbreviations: LBC1921, Lothian Birth Cohort 1921; LBC1936, Lothian Birth Cohort 1936. (For interpretation of the references to color in this figure legend, the reader is referred to the Web version of this article.)

### 3.3 Subregional brain differences

We next compared brain measures from the 2 cohorts at the level of subregions. We examined 3 different measures: subregional volume, subregional surface area, and vertex-wise cortical thickness.

#### 3.3.1 Cortical volume and surface area

The older cohort exhibited significantly lower volumes and smaller surface areas across almost all cortical regions (see Fig. 2, and Tables S2 and S3). The only regions that showed no detectable differences were the volumes of the frontal poles, the temporal poles and portions of the bilateral cingulate and right paracentral cortex, and the surface areas of the left frontal pole, the right insula, and paracentral cortex. The most pronounced effect was found in the bilateral temporal lobes on both the lateral surface (volume: all *p*-values <0.001, all *d*_*s*_ values >0.85; and surface area: all *p*-values <0.001, all *d*_*s*_ values >0.83) and medial surface (volume: all *p*-values <0.001, all *d*_*s*_ values >0.73; and surface area: all *p*-values <0.001, all *d*_*s*_ values >0.97). The left inferior temporal cortex showed the largest effect sizes of all (volume: *p* < 0.001, *d*_*s*_ = 1.63; and surface area: *p* < 0.001, *d*_*s*_ = 1.53). There were also strong group differences in the surface area of the frontal lobes (all *p*-values <0.001, all *d*_*s*_ values >0.97). Significant group effects were generally of lowest magnitude for somatosensory, motor, and cingulate regions.

#### 3.3.2 Cortical thickness

**Fig. 3:**
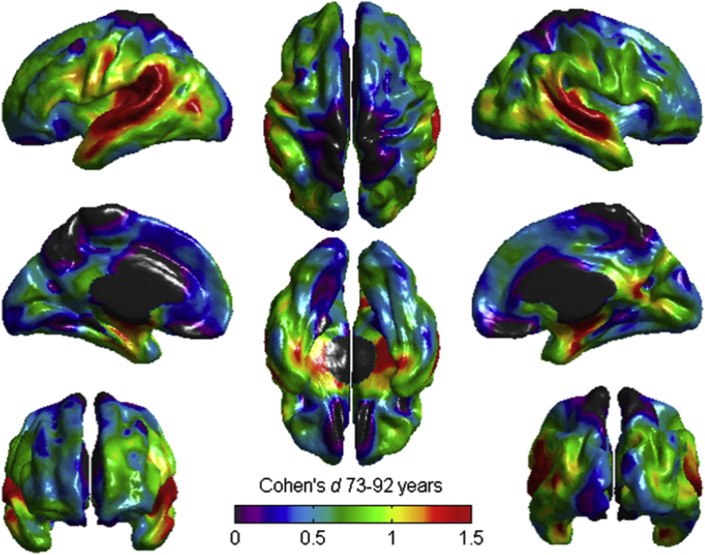
Vertex-wise differences in cortical thickness between the LBC1936 (age ∼73 years) and the LBC1921 (age ∼92 years). Warm- and gray-colored areas indicate that the greatest standardized differences between groups (*d* >1.5 SD) were localized to the superior temporal lobe. Black denotes unlabeled regions. Abbreviations: LBC1921, Lothian Birth Cohort 1921; LBC1936, Lothian Birth Cohort 1936; SD, standard deviation. (For interpretation of the references to color in this figure legend, the reader is referred to the Web version of this article.)

There were vertex-wise differences between the cohorts across most of the cortical mantle, with the strongest differences localized to the superior temporal lobe/insular cortex (Fig. 3).

#### 3.3.3 WMH location

**Fig. 4:**
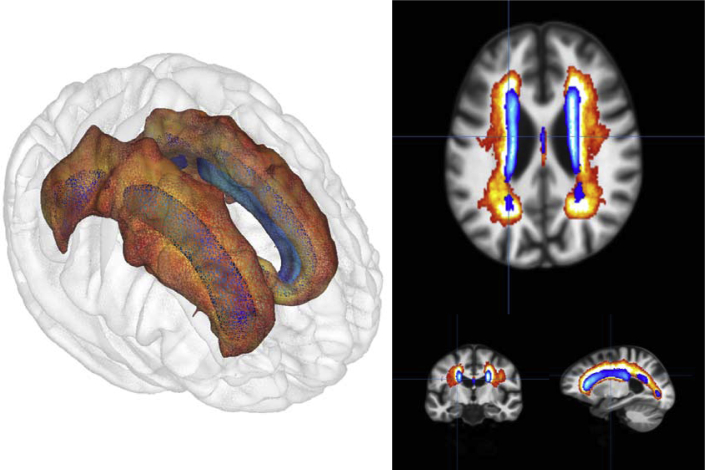
Comparison of the WMH probability maps for the age-73 LBC1936 group (blue) and age-92 LBC1921 group (red). Shown are axial, coronal, and sagittal views, along with a 3D render of the locations. Lighter colors within each cohort indicate a greater probability of WMH being found in that location. Abbreviations: LBC1921, Lothian Birth Cohort 1921; LBC1936, Lothian Birth Cohort 1936; WMH, white matter hyperintensity. (For interpretation of the references to color in this figure legend, the reader is referred to the Web version of this article.)

Probability distribution maps of WMHs comparing the 2 cohorts showed that the WMH from the younger sample (LBC1936; age 73) were mainly concentrated in the periventricular regions, particularly at the horns of the lateral ventricles. In the older sample (LBC1921, age 92), WMHs were additionally found in the deep white matter regions and were particularly abundant in the centrum semiovale. These differences are illustrated in Fig. 4.

### 3.4 Cognitive mediation models

There were substantial and significant cognitive differences between the 2 cohorts in later life (lower part of Table 1). The age-92 LBC1921 performed more poorly than the age-73 LBC1936 on all measures, particularly so for the Digit-Symbol Substitution and Logical Memory tests (*d*_*s*_-values = 2.12 and 1.58, respectively), and less so for the Verbal Fluency test (*d*_*s*_ = 0.40). There was also a substantial average difference in MMSE scores: (*d*_*s*_ = 0.97), although only 4 participants, all of them in the LBC1921, had scores below the cutoff of 24 (2 with a score of 23, 1 with 22, and 1 with 21).

**Fig. 5:**
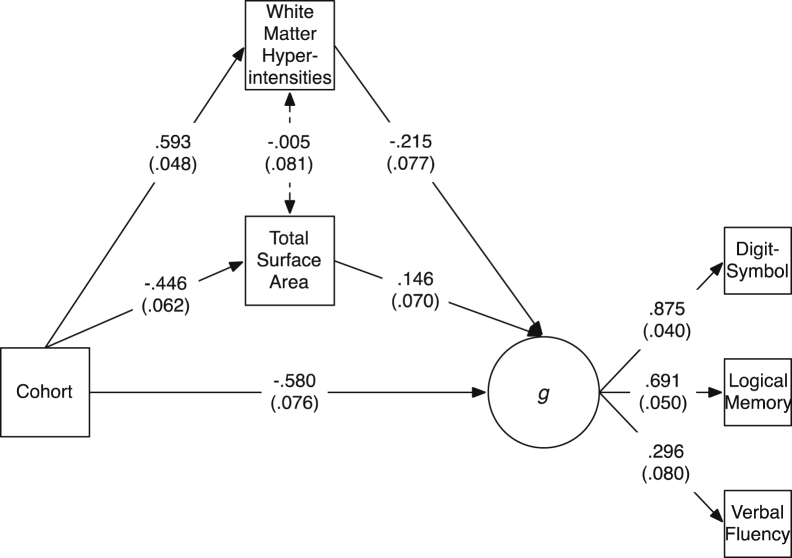
Mediation model including white matter hyperintensity volume and total surface area as mediators between cohort (age) and *g* (general cognitive ability, as indicated by 3 cognitive tests). Values are standardized path coefficients with standard errors in parentheses. The dotted line between the 2 brain variables indicates a statistically nonsignificant relation (all other relations significant at *p* < 0.05).

We tested the extent to which brain-level differences mediated the cohort (i.e., age) differences in a general cognitive ability factor (*g*) indicated by scores on the 3 normal-range cognitive tests (Digit-Symbol Substitution, Logical Memory, and Verbal Fluency; see Table S5 for a correlation matrix showing each of the brain and cognitive variables within each cohort). We first tested each of the brain variables as separate mediators. The fit statistics for each of these models, all of which had adequate or good fit to the data, are shown in Table S6. Individually, the only variables that did not significantly mediate the relation between cohort and *g* were total brain volume and mean cortical thickness (for all other variables, the bootstrapped 95% CIs did not include zero; Table S7). We then tested models including multiple previously-significant mediators to investigate which variables mediated additional variance over and above one another. In models that also included WMH volume, the mediation paths via gray matter and normal-appearing white matter volumes were reduced to nonsignificance (*β*_mediation_ [95% CIs]: 0.05 [−0.07, 0.16] and 0.01 [−0.06, 0.07], respectively). However, this was not the case for total surface area: as shown in Fig. 5, both WMH volume (*β*_mediation_ = 0.13 [0.05, 0.23]) and total surface area (*β*_mediation_ = 0.07 [0.003, 0.13]) were independently significant mediators (with the opposite direction of effects: as expected, lower WMH volume and greater surface area were related to higher *g*; this model had excellent fit to the data: *χ*^2^(6) = 6.15, *p* = 0.406, root mean square error of approximation = 0.013, comparative fit index = 0.999, Tucker-Lewis Index = 0.999, standardized root mean square residual = 0.024). Together, the 2 brain structural measurements of total surface area and volume of WMHs mediated 24.9% of the relation between cohort (age) and *g*.

### 3.5 Alternative mediation model specifications

There are a variety of alternative ways to run and specify the medication models described previously. We thank anonymous reviewers for suggesting the following alternative methods.

First, given that our sample size was relatively small, our process of including in the simultaneous model only those variables that were statistically significant may have been suboptimal: we may have been underpowered to detect true mediators of the age-cognitive association. For that reason, we ran a model including all potential brain mediators (gray matter, normal-appearing white matter, and WMH volumes along with mean cortical thickness and total surface area). This model, with 6 residual paths to take the covariance of the brain mediators into account, had adequate fit to the data (*χ*^2^(16) = 29.07, *p* = 0.023, root mean square error of approximation = 0.077, comparative fit index = 0.982, Tucker-Lewis Index = 0.960, standardized root mean square residual = 0.042). Although not all variables showed significant indirect paths, according to the bootstrapped 95% CIs (*β*_mediation_ [95% CI] for gray matter volume = −0.06 [−0.21, 0.10]; for normal-appearing white matter volume = −0.08 [−0.25, 0.04]; for WMH volume = 0.25 [0.10, 0.44]; for mean cortical thickness = 0.05 [−0.03, 0.14]; and for total surface area = 0.16 [−0.001, 0.39]), the 4 variables together mediated 43.3% of the relation between age and the *g*-factor. Thus, including all the brain mediators together, regardless of statistical significance, led to almost a doubling of the percentage of variation that was mediated.

Second, to test the extent to which the propensity score matching procedure altered the results, we ran the same mediation analyses after choosing not a propensity-score-matched comparison sample of LBC1936 participants, but a sample was chosen entirely at random from the full cohort. For every mediator except WMHs, the mediator explained a smaller percentage of the cohort-*g* relation. This indicates that, overall, the age-*g* association is somewhat more strongly mediated when the age groups are more similar in their background characteristics, possibly, because there is less noise in the estimation of the cohort-brain relation.

Third, since cross-sectional mediation models of time-dependent effects can suffer from bias (Maxwell and Cole, 2007) and because our cohort variable—intended to index age—may have confounded age with other differences between the cohorts, despite our propensity score matching approach (Raz and Lindenberger, 2011; see Discussion section for more on this potential confound), we reran the models not as mediation models, but as moderation models. We did this within a structural equation modeling framework. That is, we estimated the latent general factor of cognitive ability from the 3 tests, then allowed both WMH volume and total surface area (as in the main model presented above) to interact with age (i.e., cohort) within the model. Both of these interactions were statistically significant (*β*_age×WMH_ = −0.13, SE = 0.04, *p* < 0.001; *β*_age×totalSurfaceArea_ = 0.09, SE = 0.04, *p* = 0.02), indicating—consistent with the conclusion of the mediation models—that age changed the way in which these brain measures related to the *g*-factor.

Fourth, we ran an analysis where we controlled the multiple-mediation model (including WMH and total surface area as moderators) for vascular risk factors. This was carried to test whether the mediation by WMHs was attenuated after control for these factors (WMHs are hypothesized to be vascular related). We created a sum score of the following self-reports: smoking (1 point for being an ex-smoker, 2 points for being a current smoker), hypertension, diabetes, hypercholesterolemia, cardiovascular disease, and stroke. This “vascular risk score” ran from 0 to 6—no participants had the maximum score of 7—with an average of 2.05 (SD = 1.33). It had a zero-order correlation of *r*(164) = 0.17, *p* = 0.03 with WMH volume. We then reran the mediation model including WMH and total surface area, regressing both mediators and the outcome on the vascular risk score. This made essentially no difference to the mediation: all paths were still significant with barely changed effect sizes, and the overall percentage mediation of the cohort-*g* association from WMH and total surface area was 26.0%.

### 3.6 Analyses adjusting for the Flynn effect

In a comparison across the 2 Scottish Mental Surveys, it was noted that the MHT score of all Scottish 11-year-old participants in 1947 was on average 2.284 points higher than the score of all Scottish 11-year-old participants in 1932 (Deary et al., 2009). This reflects the well-known “Flynn effect”, which is the tendency for IQ test scores to increase generation-upon-generation (e.g., Flynn, 1987, Pietschnig and Voracek, 2015). Because the MHT score was used as a matching variable, the Flynn effect may have affected our cohort matching. For this reason, we reran the comparisons shown in Table 1 after adding 2.284 points to the MHT score of all the participants in the older (LBC1921) sample. This resulted in a slightly different sample of matched individuals from the LBC1936. The results are shown in Table S8 in the Supplementary Materials: they are nearly identical to the results from the unadjusted analysis. Probably because other variables were used for matching in addition to the MHT scores, the Flynn effect appears not to have made a substantial difference to the comparisons in this study.

### 3.7 Analyses excluding individuals with 2-year mortality and ascertained dementia

We ran 2 final sets of sensitivity analyses to test whether the main results held after removing individuals who, first, died within 2 years of their MRI scan (and thus may have been suffering from serious health conditions at the time of data acquisition) and, second, were ascertained to have a diagnosis of dementia recorded at any point before the current analyses (i.e., by August 2017). The dementia ascertainment process used a variety of methods: inspection of death certificates, inspection of electronic medical records, inspection of health status from the Scottish Morbidity Records (Information Services Division, Scotland, http://www.isdscotland.org/index.asp), or inspection of clinical reviews carried out by clinicians working alongside the LBC studies. Excluding individuals who died within 2 years of the scan reduced the sample size by 7 participants in the LBC1921 and by 4 in the LBC1936. Excluding individuals who later received a diagnosis of dementia reduced the sample size by 6 in the LBC1921 and by 7 in the LBC1936. As can be seen by comparing Tables S9 and S10, where these sensitivity results are reported, with Table 1, the broad conclusions are the same as in the overall analysis. Although the samples are small and conclusions should thus be tentative, subsequent mortality or conversion to dementia did not appear to exert a strong effect on the results reported previously.

## 4 Discussion

This propensity score matching study compared 92-year olds with a matched group of 73-year olds on a variety of structural brain measures. The 19-year difference in their ages was linked to substantial differences in brain structure. The older participants had less healthy brain tissue and higher volumes of WMHs (Fig. 1), which spread over a substantially larger area (Fig. 4). Their mean brain cortical thickness and total cortical surface area were lower. These differences were large in size, often upward of a full SD (e.g., gray matter volume was 1.74 SDs lower in the older group), and the specific differences in WMHs and cortical surface area mediated around a quarter of the association between age and general cognitive ability. The findings illustrate, using a valuable and rarely available study design, tissue- and region-general associations of age with brain structure.

Comparing our 3 subregional analyses—of subregional volumes, subregional surface areas, and vertex-wise cortical thickness—reveals broadly similar patterns of age differences. For instance, differences in frontal areas were strongly related to age, but we found that the temporal lobes showed even larger age associations. The temporal lobe is often highlighted as an area of particular importance for Alzheimer's disease (AD), with many studies noting that AD affects the temporal lobe much more prominently than normal aging (e.g., Bakkour et al., 2013, Dickerson et al., 2009, Fjell and Walhovd, 2010, Fox and Schott, 2004). The effect sizes observed here (e.g., a negative difference of *d*_*s*_ = 1.63 for the inferior temporal area across 19 years; Table S3) may serve as a baseline for future studies and comparative diagnoses; although none of our participants had a diagnosis of AD, they still showed strikingly large differences in their temporal lobes, raising the possibility that differences in this area may be a less useful indicator of AD (though see below for discussion of the results of the dementia screening test).

There were substantial differences in cognitive abilities between the samples: these were less prominent for verbal fluency but were very strong for logical memory, a measure of verbal declarative memory, and even more so for digit-symbol substitution, a measure of cognitive processing speed (Salthouse, 1996). These age differences were reflected in a large association of cohort (in this study, a strong proxy for age) with the latent *g*-factor of cognitive ability in the mediation model (Fig. 4). The healthy brain tissue volumes no longer mediated significant amounts of variance after the volume of unhealthy tissue (WMHs) was included. In a review of age-brain-cognitive relations that included 254 results from mediation analyses, Salthouse (2011) noted that there was little replicable evidence for healthy tissue volume mediating age-cognitive relations; our results would appear to be consistent with this conclusion, though this only became clear after the variable of WMH volume was added to the model.

As we have found in previous longitudinal research (Ritchie et al., 2015a), the extent of WMHs, which our lesion-mapping analysis also showed to be far larger in the older participants, appears to be the best brain structural indicator of cognitive health, at least of those considered here. Again, our results are consistent with the conclusion drawn in the review of Salthouse (2011), who concluded that WMHs mediated the age-related variance in many different cognitive tests (see also Rabbitt et al., 2007); on the basis of our results, where WMHs significantly mediated the age relation with general cognitive ability (*g*), we would tend to agree; however, we were unable to go further and examine specific domains of cognitive ability because we only had available 3 cognitive tests that were taken by participants in both cohorts. Consistent with some of our previous research in the LBC1936 cohort (Dickie et al., 2016b, Wardlaw et al., 2014), the vascular risk factors measured here, such as hypertension, smoking, diabetes, and hypercholesterolemia, accounted for only a very small proportion of the variance in brain and cognitive variables, implying that we may have to look elsewhere for health and lifestyle predictors of brain and cognitive differences in older age.

One nonvolumetric measure independently mediated age variance in the *g*-factor in addition to WMHs: total cortical surface area. Cortical thickness, on the other hand, was no longer significant in the mediation model after inclusion of WMHs. This is despite it making incremental contributions to explaining *g* level beyond hyperintensities in a previous article that used the full sample of LBC1936 participants, all at the age of 73 years (Ritchie et al., 2015b). There was, however, a good reason to predict that cortical thickness and surface area would have separable effects: not only were they weakly and nonsignificantly correlated in both the present cohorts (see Table S5), but previous longitudinal research has found them to age on distinct trajectories (Hogstrom et al., 2013, Storsve et al., 2014). Genetically informative studies have also found them to be under dissociable genetic influence (Panizzon et al., 2009; see also Eyler et al., 2012, Winkler et al., 2010). Surface area and cortical thickness have also been shown to have different relations with intelligence during development (Schnack et al., 2015) and with lifetime cognitive aging in the LBC1936 (Cox et al., 2017). In their discussion of this issue, Fjell et al. (2014) note that we currently have only a poor understanding of the reasons underlying this apparent dissociation of surface area and cortical thickness (though see Seldon, 2005, for 1 speculative mechanism regarding the “stretching” of the cortex during its development).

Overall, the effect size of the mediation by WMHs and surface area (26.4% of the cohort-*g* association) was substantial, but it leaves a large portion of the relation between age and cognitive ability unaccounted-for (this was still the case even in our alternative specification with all potential mediators, even nonsignificant ones, which resulted in a mediation percentage of 43.3%). Different or more detailed brain measurements, in larger samples so as to increase statistical power to detect smaller significant contributions, are required. Measures of the brain's white matter microstructure, whether in terms of tractography (e.g., Clayden et al., 2011; which was taken in both cohorts here but was not comparable because of the lower-resolution diffusion data obtained for the older cohort) or connectomics (which can also be examined using functional imaging; Bullmore and Bassett, 2011), as well as newer measures such as cortical complexity (Madan and Kensinger, 2016), are likely to have a role to play in explaining further portions of the age-cognitive relation (see e.g., Hakun et al., 2015, Ritchie et al., 2015c). Note that more complex mediation models, including other processes that may contribute to cognitive decline via their effects on the brain, such as age- and retirement-related declines in socioeconomic position or social resources and corresponding increases in loneliness (e.g., Shankar et al., 2013) and late-life depression (e.g., Panza et al., 2010), should be specified in future, beyond the relatively simple models considered here.

It would also be of interest, in a larger sample, to extend our mediation analyses, which only used broad measures from across the brain, to the subregional context, investigating which brain subregions (and which measures of them) mediate the largest proportion of the relation between age and cognitive ability. For example, we might predict that the aging of regions identified as being particularly relevant to general intelligence differences in the parieto-frontal integration theory model (Jung and Haier, 2007) would be prominent in explaining cognitive decline. Our results implied that the regions in the temporal lobe, as well as frontal areas, may also play a substantial role, thus providing only mixed support for this theory. However, we did not have a sample size large enough to run a mediation analysis that would have provided an appropriate theoretical test. Ultimately, a detailed picture of brain changes—and the potential influences on them—will feed in to the development of theories of cognitive aging such as the “STAC” model (Reuter-Lorenz and Park, 2014).

### 4.1 Strengths and limitations

The strengths of the study include the availability of our 2 narrow-age samples without (self-reported) dementia, one of which was in the 10th decade of life. Importantly, both samples had data on the same test of general intelligence, taken at the same age in childhood. All of the later-life cognitive and health measures were also taken comparably, and the same MRI scanner was used for all participants' scans, albeit with small differences in image resolution arising from the necessity to provide an imaging protocol of ∼30 minutes suitable for scanning participants in their 90s (LBC1921). The propensity score matching technique allowed us to select 3 similar participants from the larger LBC1936 sample for each LBC1921 participant.

The present study also includes a number of limitations that may produce biases in the results and affect their generalizability to other populations of older individuals. One important limitation of the study is the sample size of the older (LBC1921) group: we may not have had sufficient power to detect significant differences in the matching variables (though the absolute effect sizes of these differences were all relatively small). The small sample will have reduced the precision of our estimates. However, as noted previously, it is rare to have a sample of individuals with MRI data who have all reached the age of 92 years, and they were each matched with 3 members of the younger, age-73 cohort. The small sample also prevented us from examining differences in the distributions of the variables as well as in the means: future research with larger samples should test for variance differences in older participants, as was recently found for white matter diffusion measures within a large cross-sectional sample (e.g., Cox et al., 2016). Only having 2 age groups prevented us from fitting more complex, potentially nonlinear, models of brain and cognitive change with age.

Sample selectivity is also an important issue. Particularly for the older group, the fact that the participants attended for testing and MRI scanning itself implies that they were likely to be healthier than the average member of the general older-age population. This means that we will not have included individuals with more severe illness and frailty, and will potentially have underestimated the effect sizes we report here. Relatedly, the older sample—who were, at 92 years, substantially older than the country's average life expectancy—may also represent a group of individuals who are particularly robust to pathologies of aging, making them less generalizable to older people in general. Nevertheless, more individuals in the LBC1921 group than the LBC1936 group showed possible mild cognitive impairment or dementia according to the MMSE, despite not reporting having received a formal diagnosis of dementia. Although the number of participants who were below the cutoff (4 of the total 42) was small and none were substantially below the cutoff (lowest score = 21), it should be taken as an indication that aging-related pathologies may have been more commonly present in the older sample, potentially reducing the proportion of the neuroanatomical and cognitive differences found here that were entirely due to the normal aging process. Our subsequent dementia ascertainment process (which is ongoing, and may not have picked up all individuals with a diagnosis of dementia) indicated that some of the individuals who did not self-report dementia at the time of our study may subsequently have converted to pathological status. However, this did not appear to have strong effects on the main analyses, as shown in Tables S9 and S10.

A further limitation concerns cohort effects. As we have previously noted (Deary and Ritchie, 2016), the older cohort were adults by the time of the Second World War and experienced fewer years of life with free health care available on the United Kingdom's National Health Service (which was founded in 1948). The potential effects of these differences, combined with those of a large number of other social, technological, and medical advances across the 20th century, are near-impossible to control for. Naturally, all studies comparing older and younger groups suffer from similar limitations, though we were fortunate in having high-quality data on how the participants performed cognitively in childhood to provide a baseline for comparison. Nevertheless, the known problems with using cross-sectional data to infer longitudinal processes such as aging (Raz and Lindenberger, 2011) do apply here, despite the matching on childhood ability and other variables. We also acknowledge that there are a wide variety of methods for testing the significance of “indirect” paths in mediation models: we chose bias-corrected bootstrapped CIs, noted by Hayes and Scharkow (2013) as having relatively higher power than other tests, but as those authors discuss in detail, other methods exist each with their own strengths and weaknesses.

We used a validated segmentation procedure and individually checked all tissue masks for each subject in both cohorts to minimize the effect of measurement error. However, some degree of error (for instance, due to motion, registration, or image resolution) will always be present in quantitative measurements of brain tissues. Finally, we did not quantitatively measure skull thickening in these groups or control for its influence: this is likely to occur between the ages of 73 and 92 years (Aribisala et al., 2014), meaning our use of intracranial volume as a proxy for maximal brain volume is less reliable in the older sample.

### 4.2 Conclusion

Cross-sectional comparisons of aging are often limited by the lack of knowledge of how similar the participants were earlier in life. Here, we were able partly to avoid this limitation by matching our participants on several background characteristics, including childhood cognitive ability (a valuable predictor of various important late-life outcomes). Examining differences in several brain measures between matched narrow-age groups aged 73 and 92 years, we observed neurostructural differences that were often very large in size. We found that 2 of the neuroanatomical measures, WMHs and total cortical surface area, mediated one quarter of the relation between age and general cognitive ability. Although the older sample in particular was small, our results provide a measure of what can be expected neurostructurally across this period of life from a unique perspective and raise the question of which other neuroimaging measures might more fully mediate the negative relation between age and cognitive ability.

## Disclosure statement

The authors have no actual or potential conflicts of interest.
