# Supplementary material for: Brain structural differences between 73- and 92-year olds matched for childhood intelligence, social background, and intracranial volume
Source: Neurobiol Aging. 2018 Feb;62:146–58. doi: 10.1016/j.neurobiolaging.2017.10.005 (PMC5759896; doi:10.1016/j.neurobiolaging.2017.10.005)
Supplement: Supplementary Material [file mmc1.docx]

Supplementary Material for:

**Brain structural differences between 73- and 92-year olds matched for childhood intelligence, social background, and intracranial volume:**

**The Lothian Birth Cohorts**

Stuart J. Ritchie, David Alexander Dickie et al.

*Centre for Cognitive Ageing and Cognitive Epidemiology*

*The University of Edinburgh*

**Supplementary Tables**

*Table S1.* Results of the test of factorial measurement invariance across the two groups (age ~73 and age ~92).

| Type of invariance | *χ*^2^ (df) | Δ*χ*^2^ (*p*-value) | AIC | BIC |
| --- | --- | --- | --- | --- |
| Configural | 0.00 (0) | - | 3359.5 | 3414.8 |
| Weak | 2.18 (2) | 2.18 (.33) | 3357.7 | 3406.8 |
| Strong | 6.70 (4) | 4.51 (.10) | 3358.2 | 3401.2 |
| Strict | 14.19 (7) | 7.48 (.06) | 3359.7 | 3393.5 |

*Note.* Terms (“configural”, “weak”, etc) are from Widaman et al. (2010), cited in the manuscript. Configural invariance = same pattern of fixed and free loadings across groups; weak invariance = factor loadings fixed to equality across groups; strong invariance = factor loadings and observed variable intercepts fixed to equality across groups; strict invariance = factor loadings, observed variable intercepts, and observed variable variances fixed to equality across groups. Δ*χ*^2^ and *p*-value refer to a comparison with the model in the row above. AIC = Akaike Information Criterion; BIC = Bayesian Information Criterion.

*Table S2.* Coefficients from the logistic regression model run to estimate the propensity scores. The dependent variable was membership in either the LBC1921 or LBC1936 study (the logistic regression family was binomial).

| Variable | Estimate | SE | *z* | *p* |
| --- | --- | --- | --- | --- |
| Age 11 MHT | 0.07 | 0.02 | 4.24 | 2.20×10^–05^ |
| Father’s SES | –0.13 | 0.18 | –0.74 | .46 |
| Achieved SES | 1.33 | 0.22 | 5.94 | 2.90×10^–09^ |
| ICV | –7.51×10^–06^ | 1.83×10^–06^ | –4.11 | 3.98×10^–05^ |
| Sex | –1.95 | 0.53 | –3.71 | 2.09×10^–04^ |

Note: MHT = Moray House Test score; SES = Socioeconomic status; ICV = intracranial volume.

*Table S3.* Differences between the LBC1936 (age ~73) and the LBC1921 (age ~92) in brain subregional volumes, presented in descending order of effect size. These results correspond to the diagrams shown in Figure 2A.

| Brain subregion | Hemisphere | *p* | Corrected *p* | *d_s_* |
| --- | --- | --- | --- | --- |
| Inferior temporal | Left | 1.27×10^–12^ | 6.86×10^–11^ | 1.63 |
| Superior temporal gyrus | Left | 1.12×10^–09^ | 3.02×10^–08^ | 1.34 |
| Inferior temporal | Right | 2.50×10^–09^ | 3.38×10^–08^ | 1.33 |
| Middle temporal | Left | 2.20×10^–09^ | 3.38×10^–08^ | 1.26 |
| Entorhinal | Left | 1.74×10^–08^ | 1.88×10^–07^ | 1.21 |
| Middle temporal | Right | 2.35×10^–08^ | 2.12×10^–07^ | 1.19 |
| Fusiform | Left | 3.92×10^–08^ | 3.02×10^–07^ | 1.15 |
| Inferior Frontal Gyrus | Left | 4.75×10^–08^ | 3.21×10^–07^ | 1.14 |
| Parahippocampal | Right | 1.96×10^–07^ | 1.18×10^–06^ | 1.10 |
| Fusiform | Right | 4.24×10^–07^ | 1.91×10^–06^ | 1.07 |
| Lateral Orbitofrontal | Left | 2.72×10^–07^ | 1.47×10^–06^ | 1.05 |
| Entorhinal | Right | 9.45×10^–07^ | 3.40×10^–06^ | 1.04 |
| Supramarginal | Right | 9.20×10^–07^ | 3.40×10^–06^ | 1.01 |
| Precentral | Left | 1.45×10^–06^ | 4.89×10^–06^ | 1.00 |
| Lateral Orbitofrontal | Right | 1.56×10^–06^ | 4.96×10^–06^ | 0.99 |
| Inferior Parietal | Left | 9.39×10^–07^ | 3.40×10^–06^ | 0.99 |
| Lateral Occipital | Right | 3.57×10^–06^ | 1.07×10^–05^ | 0.96 |
| Superior Parietal | Left | 4.29×10^–06^ | 1.22×10^–05^ | 0.94 |
| Isthmus Cingulate | Left | 5.16×10^–06^ | 1.39×10^–05^ | 0.91 |
| Lateral Occipital | Left | 1.21×10^–05^ | 3.11×10^–05^ | 0.87 |
| Medial Orbitofrontal | Left | 1.51×10^–05^ | 3.71×10^–05^ | 0.86 |
| Inferior Frontal Gyrus | Right | 2.04×10^–05^ | 4.79×10^–05^ | 0.86 |
| Superior Temporal Gyrus | Right | 4.59×10^–05^ | 1.01×10^–04^ | 0.85 |
| Dorsolateral Prefrontal Cortex | Right | 6.73×10^–05^ | 1.40×10^–04^ | 0.82 |
| Dorsolateral Prefrontal Cortex | Left | 7.63×10^–05^ | 1.47×10^–04^ | 0.78 |
| Precuneus | Left | 7.41×10^–05^ | 1.47×10^–04^ | 0.77 |
| Inferior Parietal | Right | 1.32×10^–04^ | 2.46×10^–04^ | 0.77 |
| Postcentral | Right | 1.61×10^–04^ | 2.90×10^–04^ | 0.76 |
| Precentral | Right | 2.15×10^–04^ | 3.63×10^–04^ | 0.74 |
| Parahippocampal | Left | 2.03×10^–04^ | 3.54×10^–04^ | 0.73 |
| Medial Occipital | Right | 4.57×10^–04^ | 7.48×10^–04^ | 0.70 |
| Superior Parietal | Right | 5.93×10^–04^ | 9.39×10^–04^ | 0.69 |
| Medial Occipital | Left | 4.69×10^–05^ | 1.01×10^–04^ | 0.68 |
| Postcentral | Left | 6.09×10^–04^ | 9.39×10^–04^ | 0.67 |
| Precuneus | Right | 1.08×10^–03^ | .002 | 0.64 |
| Medial Orbitofrontal | Right | 2.64×10^–03^ | .004 | 0.59 |
| Supramarginal | Left | 3.49×10^–07^ | 1.71×10^–06^ | 0.58 |
| Rostral Anterior Cingulate | Right | .01 | .01 | 0.55 |
| Caudal Middle Frontal | Left | .004 | .01 | 0.55 |
| Isthmus Cingulate | Right | .01 | .01 | 0.53 |
| Insula | Left | .01 | .01 | 0.53 |
| Posterior Cingulate | Right | .01 | .02 | 0.48 |
| Paracentral | Left | .02 | .02 | 0.44 |
| Rostral Anterior Cingulate | Left | .03 | .04 | 0.42 |
| Caudal Middle Frontal | Right | .04 | .04 | 0.40 |
| Insula | Right | .04 | .05 | 0.39 |
| Frontal Pole | Right | .05 | .06 | 0.38 |
| Temporal Pole | Right | .08 | .09 | 0.35 |
| Frontal Pole | Left | .09 | .11 | 0.32 |
| Caudal Anterior Cingulate | Right | .13 | .14 | 0.29 |
| Temporal Pole | Left | .16 | .17 | 0.27 |
| Posterior Cingulate | Left | .21 | .22 | 0.23 |
| Paracentral | Right | .39 | .39 | 0.16 |
| Caudal Anterior Cingulate | Left | .63 | .63 | 0.09 |

Note: Corrected *p*-values are after False Discovery Rate correction.

*Table S4.* Differences between the LBC1936 (age ~73) and the LBC1921 (age ~92) in brain subregional surface areas, presented in descending order of effect size. These results correspond to the diagrams shown in Figure 2B.

| Brain subregion | Hemisphere | *p* | Corrected *p* | *d_s_* |
| --- | --- | --- | --- | --- |
| Inferior Temporal | Left | 2.96×10^–11^ | 9.05×10^–10^ | 1.53 |
| Inferior Temporal | Right | 3.35×10^–11^ | 9.05×10^–10^ | 1.50 |
| Middle Temporal | Right | 3.96×10^–10^ | 7.13×10^–09^ | 1.37 |
| Fusiform | Right | 7.00×10^–10^ | 9.45×10^–09^ | 1.36 |
| Middle Temporal | Left | 1.14×10^–09^ | 1.23×10^–08^ | 1.32 |
| Lateral Orbitofrontal | Left | 3.15×10^–08^ | 2.43×10^–07^ | 1.19 |
| Fusiform | Left | 4.91×10^–08^ | 3.24×10^–07^ | 1.16 |
| Medial Orbitofrontal | Left | 5.40×10^–08^ | 3.24×10^–07^ | 1.16 |
| Precuneus | Left | 2.64×10^–08^ | 2.38×10^–07^ | 1.15 |
| Superior Temporal Gyrus | Left | 1.25×10^–07^ | 5.67×10^–07^ | 1.15 |
| Parahippocampal | Right | 1.01×10^–07^ | 5.45×10^–07^ | 1.12 |
| Inferior Frontal Gyrus | Right | 1.26×10^–07^ | 5.67×10^–07^ | 1.10 |
| Dorsolateral Prefrontal Cortex | Right | 3.29×10^–07^ | 1.17×10^–06^ | 1.09 |
| Inferior Frontal Gyrus | Left | 3.47×10^–07^ | 1.17×10^–06^ | 1.08 |
| Lateral Orbitofrontal | Right | 2.62×10^–07^ | 1.04×10^–06^ | 1.08 |
| Medial Occipital | Left | 2.70×10^–07^ | 1.04×10^–06^ | 1.07 |
| Medial Orbitofrontal | Right | 6.14×10^–07^ | 1.79×10^–06^ | 1.06 |
| Entorhinal | Left | 5.37×10^–07^ | 1.71×10^–06^ | 1.04 |
| Entorhinal | Right | 6.30×10^–07^ | 1.79×10^–06^ | 1.01 |
| Medial Occipital | Right | 2.07×10^–06^ | 5.59×10^–06^ | 0.99 |
| Dorsolateral Prefrontal Cortex | Left | 3.04×10^–06^ | 7.66×10^–06^ | 0.97 |
| Parahippocampal | Left | 3.12×10^–06^ | 7.66×10^–06^ | 0.97 |
| Superior Parietal | Left | 4.18×10^–06^ | 9.41×10^–06^ | 0.96 |
| Rostral Anterior Cingulate | Right | 4.14×10^–06^ | 9.41×10^–06^ | 0.96 |
| Supramarginal | Left | 8.12×10^–06^ | 1.69×10^–05^ | 0.90 |
| Inferior Parietal | Left | 1.22×10^–05^ | 2.44×10^–05^ | 0.88 |
| Lateral Occipital | Left | 1.84×10^–05^ | 3.55×10^–05^ | 0.86 |
| Lateral Occipital | Right | 5.74×10^–06^ | 1.24×10^–05^ | 0.86 |
| Superior Parietal | Right | 4.09×10^–05^ | 7.36×10^–05^ | 0.84 |
| Superior Temporal Gyrus | Right | 6.92×10^–05^ | 1.13×10^–04^ | 0.83 |
| Isthmus Cingulate | Left | 2.23×10^–05^ | 4.15×10^–05^ | 0.82 |
| Precentral | Left | 5.81×10^–05^ | 1.00×10^–04^ | 0.80 |
| Supramarginal | Right | 5.95×10^–05^ | 1.00×10^–04^ | 0.80 |
| Inferior Parietal | Right | 1.43×10^–04^ | 2.26×10^–04^ | 0.76 |
| Posterior Cingulate | Right | 2.06×10^–04^ | 3.17×10^–04^ | 0.73 |
| Precuneus | Right | 2.25×10^–04^ | 3.38×10^–04^ | 0.73 |
| Posterior Cingulate | Left | 3.03×10^–04^ | 4.37×10^–04^ | 0.71 |
| Caudal Middle Frontal | Right | 3.08×10^–04^ | 4.37×10^–04^ | 0.71 |
| Rostral Anterior Cingulate | Left | 5.42×10^–04^ | 7.50×10^–04^ | 0.68 |
| Post-central | Right | 6.84×10^–04^ | 9.24×10^–04^ | 0.67 |
| Caudal Middle Frontal | Left | .001 | .001 | 0.63 |
| Post-central | Left | .002 | .003 | 0.62 |
| Insula | Left | .003 | .003 | 0.58 |
| Frontal Pole | Right | .003 | .004 | 0.58 |
| Caudal Anterior Cingulate | Left | .005 | .01 | 0.54 |
| Precentral | Right | .01 | .01 | 0.51 |
| Temporal Pole | Left | .01 | .02 | 0.48 |
| Paracentral | Left | .02 | .02 | 0.46 |
| Isthmus Cingulate | Right | .02 | .03 | 0.44 |
| Caudal Anterior Cingulate | Right | .03 | .03 | 0.42 |
| Temporal Pole | Right | .04 | .04 | 0.41 |
| Frontal Pole | Left | .04 | .05 | 0.38 |
| Insula | Right | .07 | .07 | 0.34 |
| Paracentral | Right | .08 | .08 | 0.33 |

Note: Corrected *p*-values are after False Discovery Rate correction.

*Table S5.* Correlation matrix showing relations between brain variables and cognitive tests for the LBC1921 members (below diagonal) and the matched LBC1936 members (above diagonal; see Table 1 in the main document for the *n* for each individual test).

| Variable | 1 | 2 | 3 | 4 | 5 | 6 | 7 | 8 | 9 | 10 | 11 |
| --- | --- | --- | --- | --- | --- | --- | --- | --- | --- | --- | --- |
| 1. Total brain volume | - | .93^***†^ | .90^***^ | −.14 | .33^***^ | .90^***^ | .19^*^ | −.02 | .09 | .23^*^ | .18^*^ |
| 2. Grey matter volume | .82^***†^ | - | .77^***^ | −.24^**^ | .41^***†^ | .82^***^ | .18^*^ | −.004 | .07 | .22^*^ | .17 |
| 3. Normal-appearing white matter volume | .90^***^ | .74^***^ | - | −.42^***^ | .23^*^ | .84^***^ | .25^**^ | −.04 | .16 | .30^*^ | .16 |
| 4. White matter hyperintensity volume | −.09 | −.39^**^ | −.40^**^ | - | −.06 | −.13 | −.26 | .01 | −.18^*^ | −.33^**^ | −.03 |
| 5. Mean cortical thickness (mm) | .08 | .04^†^ | −.07 | .18 | - | .09 | .19^*†^ | −.05 | .02 | .20 | .10 |
| 6. Total surface area (cm^2^) | .84^***^ | .68^***^ | .75^***^ | .15 | .09 | - | .17 | .04 | .08 | .23 | .19^*^ |
| 7. Digit-Symbol Substitution | .13 | .36^*^ | .20 | −.32^*^ | −.31^†^ | .23 | - | .25^**^ | .26^**^ | .63^**^ | .44^***^ |
| 8. Logical Memory | .05 | .10 | .05 | −.14 | −.11 | .04 | .50^***^ | - | .16 | .40^**†^ | .39^***^ |
| 9. Verbal Fluency | −.01 | .01 | -.02 | −.22 | −.31^*^ | -.05 | .29 | .21 | - | .40^**^ | .40^***^ |
| 10. *g*-factor | .14 | .30 | .10 | −.26 | −.06 | .20 | .73^**^ | .83^**†^ | .34^*^ | - | .86^***†^ |
| 11. Age 11 intelligence | −.19 | .02 | −.16 | −.08 | −.17 | −.06 | .24 | .09 | .40^**^ | .26^†^ | - |

Note: * = *p* < .05; ** = *p* < .01; *** = *p* < .001; † = correlation is statistically significantly different (*p* < .05) from the equivalent correlation in the other sample, by use of the ‘r.test’ function in the ‘psych’ package for R (Revelle, 2016); *g*-factor = general factor of cognitive ability estimated from the three cognitive tests; the correlations between the *g*-factor and the three cognitive tests are their standardized loadings within each cohort.

*Table S6.* Single-mediator model results. The three paths in each model—cohort-mediator, mediator-*g*, and cohort-*g* (where *g* is the general factor of cognitive ability)—each have a standardized path coefficient (*β*) and a standard error. The effect size of the mediation is expressed as a percentage of the cohort-*g* effect in a model without the mediating paths.

| Mediator | *β*_cohort-mediator_ (SE) | *β*_mediator-_*_g_* (SE) | *β*_cohort-_*_g_* (SE) | *β*_mediation_ (bootstrapped 95% CI) | % mediation | % mediation (random-selection model) |
| --- | --- | --- | --- | --- | --- | --- |
| Total brain volume | .21 (.08)^**^ | .11 (.07) | .74 (.05)^***^ | .02 (-.01, .07) | 2.9% | 2.2% |
| Grey matter volume | .52 (.05)^***^ | .16 (.08)^*^ | .68 (.06)^***^ | .08 (.01, .17) | 10.8% | 7.5% |
| Normal-appearing white matter volume | .33 (.07)^***^ | .15 (.07)^*^ | .71 (.05)^***^ | .05 (.004, .11) | 6.6% | 4.4% |
| White matter hyperintensity volume | -.59 (.05)^***^ | -.22 (.08)^**^ | .65 (.07)^***^ | .13 (.05, .24) | 16.9% | 17.2% |
| Mean cortical thickness | .40 (.07)^***^ | .08 (.08) | .73 (.06)^***^ | .03 (-.03, .10) | 4.1% | 2.2% |
| Total surface area | .47 (.06)^***^ | .14 (.07) | .72 (.06)^***^ | .06 (.01, .13) | 8.3% | 6.7% |

Note: * = *p* < .05; ** = *p* < .01; *** = *p* < .001; the ‘random model’ mediation percentages come from models where, instead of propensity score matching, the younger (LBC1936) participants for the comparison were chosen at random – the number given is the average of five such random models per mediator.

*Table S7.* Comparisons between LBC1921 (*n* = 42; age ~92) and propensity-score matched LBC1936 (*n* = 126; age ~73) participants on each brain measure, for the sample who had data on all matching variables, after adjusting for potential Flynn Effects (generational increases) in the age 11 Moray House Test score. This table is the equivalent of Table 1 in the main document. Note that the means and sample sizes for the LBC1921 sample are identical to those in Table 1, except for the Flynn-adjusted age 11 MHT score.

| Measure category | Measure | *n* | | Sample mean (SD)/% | | Difference test | | |
| --- | --- | --- | --- | --- | --- | --- | --- | --- |
|  |  | LBC1921 | LBC1936 | LBC1921 | LBC1936 | *t* | *p* | *d_s_* |
| *Matching variables* | Father’s SES | 42 | 126 | 2.90 (1.28) | 2.92 (1.03) | −0.11 | .91 | 0.02 |
|  | Achieved SES | 42 | 126 | 2.26 (0.99) | 2.42 (0.82) | −0.94 | .35 | 0.17 |
|  | Age 11 MHT | 42 | 126 | 49.64 (10.32) | 49.86 (13.09) | −0.11 | .91 | 0.02 |
|  | ICV | 42 | 126 | 1490.91 (140.18) | 1486.51 (143.84) | 0.17 | .86 | 0.03 |
|  | Sex | 42 | 126 | 40.5% male | 41.3% male | - | 1.00 | - |
|  |  |  |  |  |  |  |  |  |
| *Brain tissue measures* | TBV (cm^3^) | 38 | 126 | 954.73 (95.17) | 1021.46 (95.68) | −3.78 | <.001 | 0.70 |
|  | GMV (cm^3^) | 37 | 126 | 404.76 (39.02) | 483.34 (49.95) | −10.07 | <.001 | 1.88 |
|  | NAWM (cm^3^) | 37 | 126 | 434.02 (70.73) | 496.31 (53.88) | −4.95 | <.001 | 0.93 |
|  | WMH (cm^3^) | 40 | 126 | 46.87 (30.42) | 12.24 (10.65) | 7.06 | <.001 | 1.28 |
|  | MCT (mm) | 37 | 85 | 2.97 (0.14) | 3.15 (0.15) | −6.22 | <.001 | 1.23 |
|  | TSA (cm^2^) | 39 | 122 | 1377.11 (164.61) | 1576.84 (146.95) | −6.76 | <.001 | 1.24 |
|  |  |  |  |  |  |  |  |  |
| *Later-life*  *cognitive tests* | Digit-Symbol | 34 | 125 | 33.32 (10.66) | 59.04 (12.45) | −12.02 | <.001 | 2.32 |
|  | Logical Memory | 42 | 126 | 9.90 (4.48) | 16.52 (3.84) | −8.57 | <.001 | 1.53 |
|  | Verbal Fluency | 42 | 126 | 38.43 (13.64) | 44.65 (12.22) | −2.63 | .01 | 0.47 |
| *Dementia screening* | MMSE | 42 | 125 | 26.90 (2.36) | 28.87 (1.31) | −5.15 | <.001 | 0.92 |

*Note:* Differences calculated using Welch’s two-sample *t*-test; for Cohen’s *d_s_*, see Lakens (2013); LBC1921 = Lothian Birth Cohort 1921; LBC1936 = Lothian Birth Cohort 1936; SES = socioeconomic status; MHT = Moray House Test, ICV = intracranial volume, TBV = total brain volume, GMV = grey matter volume, NAWM = normal-appearing white matter volume, WMH = white matter hyperintensity volume, MCT = mean cortical thickness, TSA = total cortical surface area; MMSE = Mini-Mental State Examination.

**Supplementary Figures**

*Figure S1.* Vertex-wise descriptive differences in cortical thickness between the LBC1936 (age ~73) and the LBC1921 (age ~92). Warm colored areas in the top (age ~73) and middle (age ~92) panels indicate the thinnest areas of cortex.


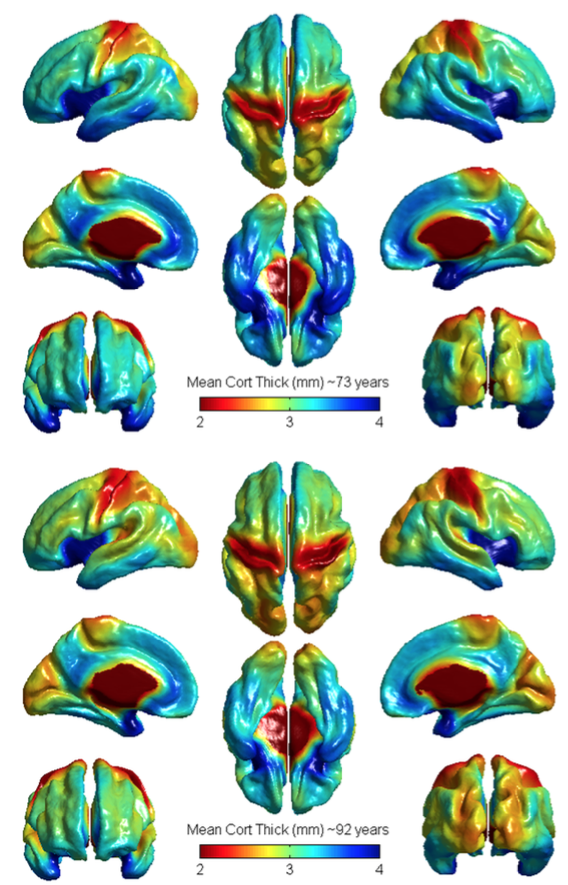


*Figure S2*. Vertex-wise statistically significant differences in cortical thickness between the LBC1936(age ~73) and the LBC1921 (age ~92). Warm areas on the left panel indicate that the highest *t*-values between groups were in the superior temporal lobe; the right panel shows that there were statistically significant negative differences between age ~73 and ~92 years in cortical thickness across the majority of the cortical mantle (yellow and orange areas).


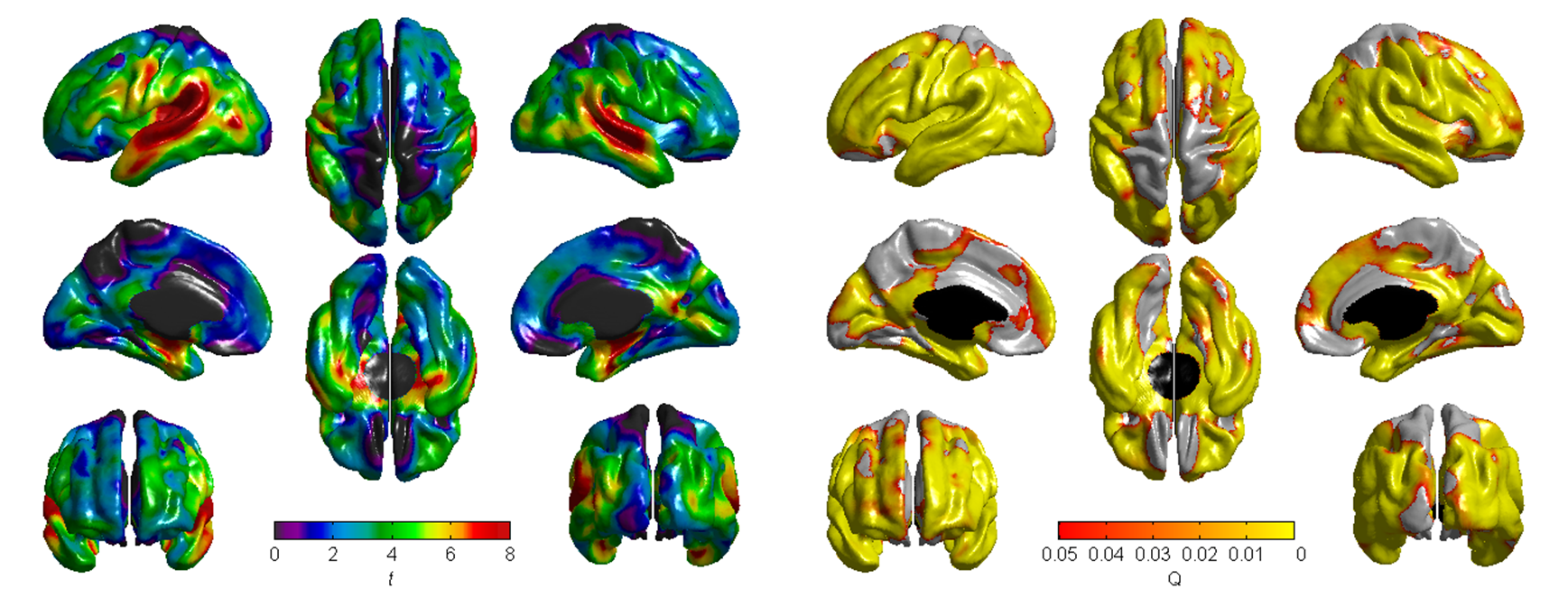


**Supplementary Reference**

Revelle, W. (2016). psych: Procedures for psychological, psychometric, and personality research. *R* package version 1.6.4. http://CRAN.R-project.org/package=psych
